# Risk factors for emotional and behavioral problems in moderately-late preterms

**DOI:** 10.1371/journal.pone.0216468

**Published:** 2019-05-02

**Authors:** Pauline J. den Haan, Marlou L. A. de Kroon, Nienke H. van Dokkum, Jorien M. Kerstjens, Sijmen A. Reijneveld, Arend F. Bos

**Affiliations:** 1 Department of Pediatrics, Division of Neonatology, Beatrix Children’s Hospital, University Medical Center Groningen, University of Groningen, Groningen, The Netherlands; 2 Department of Health Sciences, University Medical Center Groningen, University of Groningen, Groningen, The Netherlands; International Telematic University Uninettuno, ITALY

## Abstract

**Objective:**

To assess which factors, including maternal, lifestyle, pregnancy- and delivery-related, fetal and neonatal factors adjusted for socio-economic status, are related to emotional and behavioral problems in moderately-late preterm born children (MLPs; gestational age 32.0–35.9 weeks) at 4 years of age. MLPs are at greater risk of emotional and behavioral problems than full-term born children. Especially for MLPs, knowledge about factors that increase or decrease the risk of emotional and behavioral problems is scarce.

**Design and setting:**

We assessed emotional and behavioral problems in 809 MLPs between ages 41 and 49 months from the prospective community-based Longitudinal Preterm Outcome Project (LOLLIPOP), using the parent-reported Child Behavior Checklist (CBCL). We collected potential risk factors from hospital records and parental questionnaires. Univariable and multiple logistic regression analyses were applied.

**Main outcome measures:**

(Sub)clinical CBCL scores.

**Results:**

Perinatal infection increased the risk of CBCL total problem scores with an OR 2.22 (*p*<0.01). Perinatal infection, maternal smoking, and male gender increased the risk of CBCL externalizing problem scores with ORs between 1.64 and 2.46 (all *p*<0.05). Multiple birth decreased the risk of CBCL internalizing problem scores with an OR 0.63 (*p*<0.05).

**Conclusions:**

Risk factors for behavioral problems in MLPs are male gender, perinatal infection and maternal smoking, the latter two being potentially modifiable. Multiple birth is a protective factor for emotional problems in MLPs. These results suggest potential factors for targeting preventive intervention in MLPs, comprising the large majority of all preterm born children.

## Introduction

Moderately-late preterm born children (MLPs, gestational age (GA) 32.0–35.9 weeks, 85% of all preterm born children [1]) are at a 1.5 to 2.5-fold increased risk of emotional and behavioral problems compared to full-term born children (FTs, GA 38.0–41.9 weeks) [2–4]. Additionally, MLPs born healthy at birth demonstrate more emotional and behavioral problems than FTs treated at the neonatal intensive care unit (NICU) after birth [3]. These emotional and behavioral problems frequently persist in later life [5]. They affect the child’s quality of life [6], and may lead to grade retention [2], special educational needs [2,7], and numerous long-term adversities such as employment difficulties [8,9], crime, and substance abuse in adulthood [10].

For MLPs, there is only little evidence on factors that increase the risk of emotional and behavioral problems. The few factors that have been identified are admission to a NICU [3], low socioeconomic status (SES) [11], and female gender, all increasing the risk of emotional problems at 1.5 years of age [12]. Poorer longitudinal postnatal growth was not found to be associated with emotional and behavioral problems at seven years of age [13], and a British study from 2001 [7] found several factors increasing the risk of school problems in MLPs, such as male sex and postnatal discharge from the special baby care unit beyond 36 weeks postmenstrual age. In contrast, studies among both early preterm born children (EPs, GA <32 weeks) and FTs report several factors to be associated with increased risk of emotional and behavioral problems [14–19]. The factors as identified regard several domains: parental-emotional (e.g. emotional maternal and couple problems [14], maternal smoking, and poor maternal physical and mental well-being [15,20]), delivery-related (e.g. blood loss during pregnancy [16], and caesarian section [14]), and neonatal (e.g. male gender [14,17,21], and (prolonged) NICU admission [14,16,18]). Next risk factors and their effects may definitely be different for the group of MLPs. For example, in a study based on our own cohort, SES was found to have a stronger effect on the development of emotional and behavioral problems in children with a lower GA [11].

Because of the large clinical and public health consequences of emotional and behavioral problems, more insight into risk factors that are associated with these problems in this largest group of preterm born children may expedite the identification of MLPs at risk. Since early intervention on emotional and behavioral problems has proven to be effective for both FTs and preterm born children in general [22,23], new knowledge on potentially modifiable risk factors and on identification of MLPs at increased risk may be used for targeted preventive interventions. Therefore, we aimed to investigate which factors, including maternal, lifestyle, pregnancy- and delivery-related, fetal and neonatal factors increase or decrease the risk of total, externalizing, and internalizing emotional and behavioral problems in MLPs at school entry (i.e. 4 years).

## Methods

### Study design and sampling procedure

This study is part of the Longitudinal Preterm Outcome Project (LOLLIPOP) [24]. In total, thirteen Dutch Preventive Child Healthcare Centers (PCHCs) examined charts of 45,446 children (25% of a Dutch year cohort) aged 43 to 49 months, who were born in 2002 and 2003 in the three northern provinces of the Netherlands. Of those 45,446 children, 1,412 were born moderately-late preterm. Parents of 1,145 children gave written informed consent, leading to an inclusion rate of 81%. Exclusion criteria were congenital malformations or syndromes, congenital infections, and a GA that could not be verified or was outside the set range. Non-participating children more often had parents with low SES and/or non-Dutch ethnicity (both P<0.001), and were part of a multiple pregnancy less often (P<0.05) [25]. For this study, to obtain as homogeneous as possible a study sample we sampled only MLPs that joined the follow-up at school entry, had the Child Behavior Checklist (CBCL) administered between 41 and 49 months of age, and for which information on all risk factors was available (N = 809, i.e. 71% of parents willing to participate). The LOLLIPOP-study was approved by the University Medical Center Groningen review board (ISRCTN register trial number 80622320) and written informed consent was obtained from all parents. A complete flowchart of our sampling procedure has been previously reported [24].

### Data and data collection

A month prior to the last scheduled PCHC visit at age 43–49 months parents received an invitation to let their child participate in the study. Included in the invitation were information about the LOLLIPOP-study, an informed consent form, an emotional and behavioral problem questionnaire (CBCL), and a general questionnaire on familial and perinatal characteristics, all of which parents returned during their visit.

We assessed emotional and behavioral problems at age 4, using the CBCL suitable for ages 1.5–5 years. The CBCL is a parental questionnaire containing 99 problem items with ratings between not true and often/very true. It was filled out by the mother in approximately 81% of MLPs included in this study. These questions were all included in the total problems scale, and two broadband scales, internalizing (i.e. ‘emotional’) and externalizing (i.e. ‘behavioral’) problems, were computed [26]. CBCL data were dichotomized conform the manual, with cut-off scores set at ≤83% (normal), and ≥84% ((sub)clinical). The CBCL has good psychometric properties and a validated Dutch version [26–28].

Based on the literature, we assessed which factors we would analyze as potential risk factors for emotional and behavioral problems in MLPs: (1) known risk factors for emotional and behavioral problems in FTs, EPs [14–16,18,19,21], and MLPs [3,11,12], and (2) known risk factors for general developmental problems in MLPs (Table 1) [7,29,30]. Data on these maternal and lifestyle factors, pregnancy- and delivery-related factors, and fetal and neonatal factors were collected from a general questionnaire, birth registers, and medical records of both mother and child. This made it possible to cross-check information from different sources. GA was confirmed by early ultrasound measurements in >95% of cases. In other cases, GA was cross-checked against clinical estimates after birth. Data were coded following standard practices.

**Table 1:** Potential risk factors among MLP for emotional and behavioral problems within the LOLLIPOP cohort.

Potential risk factors	Prevalence		Definition of the variable
	n/N_total_	%	
**Maternal and lifestyle**			
Non-Dutch ethnicity	42/809	(5.2)	Mother not born within the Netherlands
Multiparity	284/809	(35.1)	Mother who has gone through ≥1 pregnancies
Pre-pregnancy obesity	88/767	(11.5)	BMI > 30kg/m^2^
Smoking during pregnancy	185/807	(22.9)	Any smoking during pregnancy
Maternal mental illness	13/809	(1.6)	Chronic mental illness (depression, psychosis, other)
**Pregnancy- and delivery-related**			
HELLP/pre-eclampsia	157/809	(19.4)	(Severe type of) pre-eclampsia due to placenta malfunction
Antenatal steroids	156/809	(19.3)	Completed treatment with antenatal steroids
Induced birth; fetal reasons	121/809	(15.0)	Fetal/combined fetal + maternal indication of induced delivery
pPROM	187/809	(23.1)	Prolonged premature rupture of membranes (>24 hours before delivery)
C-section	293/809	(36.2)	Primary or secondary caesarian section
Perinatal infection	119/809	(14.7)	Clinical signs of bacterial infection, of mother and/or child, or proven chorioamnionitis
**Fetal and neonatal**			
Gender	459/809	(56.7)	Male sex
Multiple birth	233/809	(28.8)	Being part of a multiple birth (twin, triplet)
SGA	72/809	(8.9)	Small for gestational age below P10 according to Dutch growth charts
Lower GA	259/809	(32.0)	Gestational age 32–33 weeks (opposed to GA 34–35 weeks)
Asphyxia	17/807	(2.1)	Asphyxia in conclusion in discharge letter
Circulatory insufficiency	24/807	(3.0)	≥1 time inotropic medication administration
Respiratory insufficiency	144/807	(17.8)	CPAP and/or assisted ventilation in delivery room for longer than initial stabilization
Caffeine treatment	89/802	(11.1)	Caffeine treatment for apnea
Hyperbilirubinemia	351/805	(43.6)	Peak bilirubin value: and/or phototherapy. GA 32-33wk: >255 μmol/L, GA 34-35wk: >340 μmol/L
Hypoglycemia	65/798	(8.1)	In first 72 hours at least one recorded plasma glucose value < 1.7 mmol/L (30 mg/dL)
Septicemia	30/806	(3.7)	Clinical symptoms and ≥1 positive blood culture test
Highest P10 length of hospital stay	75/799	(9.4)	In upper 10% of duration of hospital admission compared to peers of the same GA week
**Sociodemographic**			
SES			Socio-economic status
Low	212/808	(26.2)	≥1SD below mean
Intermediate	408/808	(50.5)	mean +/- 1SD
High	188/808	(23.3)	≥1SD above mean

Abbreviations: BMI: Body Mass Index; HELLP: Hemolysis Elevated Liver Enzymes, and Low Platelet Count; CPAP: continuous positive airway pressure; SES: Socio-economic status; based on five measurements including education of the mother, education of the father, parental income, occupation of the mother and occupation of the father.

### Statistical analyses

First, using Chi-Square Tests we assessed background characteristics of the sample in relation to the presence of dichotomized CBCL total problems. Second, using univariable logistic regression analyses we assessed the risks of increased CBCL total, internalizing, and externalizing problems for various maternal and lifestyle factors, pregnancy- and delivery-related factors, and fetal and neonatal factors. Finally, we included in the final multivariable logistic regression analysis all risk factors that had a *p*-value <0.2 in the univariable logistic regression analyses [18], and adjusted for SES based on the combination of education of the mother, education of the father, occupation of the mother, occupation of the father and parental income [11]. In this multivariable logistic regression analysis we performed a backward stepwise selection, applying backward elimination based on a statistical significance level of *p*<0.05. All analyses were performed using SPSS, version 23 (SPSS Inc., Chicago, Illinois, USA).

## Results

### Background characteristics

Prevalence rates of the potential risk factors included in our study are shown in Table 1. In our sample of MLPs, more children were male (n = 459; 56.7%), and one third were born with a GA of 32–33 weeks (n = 259, 32.0%).

### Potential risk factors with increased CBCL problem scores

Prevalence rates of CBCL problems in MLPs are shown in Table 2. Overall, 118 (14.6%) of the MLPs had CBCL total problems, 135 (16.7%) MLPs had CBCL externalizing problems, and 137 (16.9%) MLPs had CBCL internalizing problems.

**Table 2:** Risk of increased CBCL total, externalizing, and internalizing problem scores for potential risk factors at age 4: Results of univariable logistic regression analyses.

Potential risk factors	N	Total problems			Externalizing problems			Internalizing problems		
		Prevalence		*P*-value^§^	Prevalence		*P*-value^§^	Prevalence		*P*-value^§^
		n	%		n	%		n	%	
**Total:**	809	118	14.6		135	16.7		137	16.9	
**Maternal and lifestyle**										
Non-Dutch ethnicity	42	8	(19.0)	0.40	8	(19.0)	0.67	9	(21.4)	0.43
Multiparity	284	47	(16.5)	0.25	55	(19.4)	0.13#	51	(18.0)	0.57
Pre-pregnancy obesity	88	12	(13.6)	0.84	15	(17.0)	0.81	16	(18.2)	0.74
Smoking during pregnancy	185	35	(18.9)	0.053#	**43**	**(23.2)**	0.006**	38	(20.5)	0.12#
Maternal mental illness	13	3	(23.1)	0.39	**5**	**(38.5)**	0.04*	3	(23.1)	0.55
**Pregnancy- and delivery-related**										
HELLP /pre-eclampsia	157	24	(15.3)	0.78	30	(19.1)	0.37	28	(17.8)	0.74
Antenatal steroids	156	28	(17.9)	0.19#	31	(19.9)	0.24	30	(19.2)	0.40
Induced birth; fetal reasons	121	20	(16.5)	0.51	22	(18.2)	0.63	23	(19.0)	0.51
pPROM	187	29	(15.5)	0.68	31	(16.6)	0.96	39	(20.9)	0.10#
C-section	293	38	(13.0)	0.33	49	(16.7)	0.98	44	(15.0)	0.27
Perinatal infection	119	**29**	**(24.4)**	0.001**	**33**	**(27.7)**	0.001**	26	(21.8)	0.12#
**Fetal and neonatal**										
Gender	459	73	(15.9)	0.23	**91**	**(19.8)**	0.007**	73	(15.9)	0.37
Multiple birth	233	30	(12.9)	0.38	**29**	**(12.4)**	0.04*	31	(13.3)	0.08#
SGA	72	15	(20.8)	0.12#	16	(22.2)	0.19#	15	(20.8)	0.36
Lower GA	259	38	(14.7)	0.96	49	(18.9)	0.24	39	(15.1)	0.33
Asphyxia	17	3	(17.6)	0.72	3	(17.6)	0.92	5	(29.4)	0.18#
Circulatory insufficiency	24	4	(16.7)	0.77	5	(20.8)	0.59	3	(12.5)	0.56
Respiratory insufficiency	144	23	(16.0)	0.61	25	(17.4)	0.82	22	(15.3)	0.55
Caffeine treatment	89	9	(10.1)	0.197#	13	(14.6)	0.55	10	(11.2)	0.12#
Hyperbilirubinemia	351	55	(15.7)	0.42	63	(17.9)	0.38	55	(15.7)	0.37
Hypoglycemia	65	9	(13.8)	0.85	16	(24.6)	0.08#	11	(16.9)	0.98
Septicemia	30	7	(23.3)	1.18	6	(20.0)	0.63	5	(16.7)	0.96
Highest P10 length of hospital stay	75	10	(13.3)	0.74	9	(12.0)	0.26	12	(16.0)	0.83

^#^ P<0.2,

* P< 0.05,

** P<0.01.

^§^ Odds ratios (OR) and 95% confidence intervals (CI) are given in Table 3.

Abbreviations: HELLP: Hemolysis Elevated Liver Enzymes, and Low Platelet Count; C-section: caesarian section; pPROM: prolonged premature rupture of membranes; SGA: Small for Gestational Age; Lower GA: lower gestational age.

In the multivariable analyses, perinatal infection increased the risk (OR 2.22 (95% CI 1.38–3.58)) of CBCL total problems, shown in Table 3. Perinatal infection, maternal smoking during pregnancy, and male gender significantly increased the risk (OR 2.46 (95% CI 1.55–3.93), 1.64 (1.07–2.52), and 1.77 (1.19–2.65), respectively of CBCL externalizing problems. Lastly, only being part of a multiple birth significantly decreased the risk (OR 0.63 (95% CI 0.40–0.98)) of CBCL internalizing problems.

**Table 3:** Odds ratios (OR) and 95% confidence intervals (CI) of the risk of increased CBCL total, externalizing, and internalizing problem scores for potential risk factors at age 4, adjusted for socio-economic status (SES) in multivariable analyses.

Potential risk factors	Total problems				Externalizing problems				Internalizing problems			
	Univariable^§^		Multivariable^¥^		Univariable^§^		Multivariable^¥^		Univariable^§^		Multivariable^¥^	
	OR	95% CI	OR	95% CI	OR	95% CI	OR	95% CI	OR	95% CI	OR	95% CI
**Maternal and lifestyle**												
Non-Dutch ethnicity	1.41	(0.63–3.12)			1.19	(0.54–2.62)			1.36	(0.64–2.91)		
Multiparity	1.27	(0.85–1.89)			1.34	(0.92–1.95)#	**---**		1.12	(0.76–1.64)		
Pre-pregnancy obesity	0.94	(0.49–1.79)			1.08	(0.59–1.94)			1.10	(0.62–1.96)		
Smoking during pregnancy	1.54	(0.99–2.37)#	**---**		**1.77**	(1.18–2.66)**	**1.64**	(1.07–2.52)*	1.40	(0.92–2.12)#	**---**	
Maternal mental illness	1.78	(0.48–6.55)			**3.20**	(1.03–9.94)*	**---**		1.48	(0.40–5.46)		
**Pregnancy- and delivery-related**												
HELLP/pre-eclampsia	1.07	(0.66–1.74)			1.23	(0.79–1.93)			1.08	(0.68–1.71)		
Antenatal steroids	1.37	(0.86–2.18)#	**---**		1.31	(0.84–2.04)			1.22	(0.78–1.90)		
Induced birth; fetal reasons	1.19	(0.71–2.02)			1.13	(0.68–1.87)			1.18	(0.72–1.94)		
pPROM	1.01	(0.70–1.73)			0.99	(0.64–1.54)			1.41	(0.93–2.13)#	**---**	
C-section	0.81	(0.54–1.23)			1.00	(0.68–1.48)			0.80	(0.54–1.19)		
Perinatal infection	**2.18**	(1.35–3.50)**	**2.22**	(1.38–3.58)**	**2.21**	(1.41–3.48)**	**2.46**	(1.55–3.93)**	1.46	(0.90–2.36)#	**---**	
**Fetal and neonatal**												
Gender	1.28	(0.86–1.91)			**1.72**	(1.16–2.54)**	**1.77**	(1.19–2.65)**	0.85	(0.58–1.22)		
Multiple birth	0.82	(0.53–1.28)			**0.63**	(0.41–0.98)*	**---**		0.68	(0.44–1.05)#	**0.63**	(0.40–0.98)*
SGA	1.62	(0.88–2.97)#	**---**		1.48	(0.82–2.68)#	**---**		1.33	(0.73–2.42)		
Lower GA	1.01	(0.67–1.53)			1.26	(0.86–1.85)			0.82	(0.55–1.23)		
Asphyxia	1.26	(0.36–4.45)			1.07	(0.30–3.77)			2.08	(0.72–5.99)#	**---**	
Circulatory insufficiency	1.17	(0.39–3.50)			1.32	(0.49–3.60)			0.69	(0.20–2.35)		
Respiratory insufficiency	1.14	(0.69–1.87)			1.06	(0.66–1.70)			0.86	(0.52–1.41)		
Caffeine treatment	0.62	(0.30–1.28)#	**---**		0.83	(0.45–1.54)			0.58	(0.29–1.16)#	**---**	
Hyperbilirubinemia	1.18	(0.79–1.74)			1.18	(0.81–1.71)			0.84	(0.58–1.23)		
Hypoglycemia	0.93	(0.45–1.94)			1.70	(0.94–3.09)#	**---**		0.99	(0.50–1.95)		
Septicemia	1.82	(0.76–4.35)			1.25	(0.50–3.13)			0.98	(0.37–2.60)		
Highest P10 length of hospital stay	0.89	(0.44–1.78)			0.66	(0.32–1.36)			0.93	(0.49–1.78)		

^#^ P<0.2;

* P< 0.05;

** P<0.01;

---signifies: ‘did not remain in the model with p<0.05 after backward selection’.

^§^ Only one potential risk factor included in the model.

^¥^ All variables with P<0.2 in univariable analyses were included in the multivariable model, adjusted for socio-economic status.

Abbreviations: HELLP: Hemolysis Elevated Liver Enzymes, and Low Platelet Count; C-section: caesarian section; pPROM: prolonged premature rupture of membranes; SGA: Small for Gestational Age; Lower GA: lower gestational age.

## Discussion

Our study demonstrated that in MLPs perinatal infection increased the risk of CBCL total problems, and particularly the risk of CBCL externalizing problems, at school entry. The risk of CBCL externalizing problems was also higher when the mother smoked during pregnancy, and for males. Being part of a multiple birth decreased the risk of CBCL internalizing problems.

We found perinatal infections in particular to be associated with a more than twofold increased risk of behavioral problems. In our study, perinatal infection refers to a suspected bacterial infection of the mother and/or child based on clinical signs, or proven chorioamnionitis. Likewise, Lee et al. (2015) [31] found bacterial infection during pregnancy to be associated with autism spectrum disorders in the newborn child. However, the findings of other studies contradicted ours regarding the association between chorioamnionitis and emotional and behavioral problems [32]. This discrepancy may be explained by the clinical signs of infection in the mother and/or child which we included in the definition of our risk factor ‘perinatal infection’. Clinically diagnosed perinatal and neonatal infections during delivery are the result of systemic inflammatory responses (cytokines, free radicals) [33] and altered feto-placental and neonatal hemodynamics. We hypothesize that these systemic inflammatory and hemodynamic responses lead to structural brain injury [34], which may in turn be responsible for impaired behavioral functioning at later ages in MLPs. This hypothesized pathogenetic pathway is supported by our finding that prolonged premature rupture of the membranes, which often goes without a systemic inflammatory response, was not associated with increased risk.

We found that maternal smoking during pregnancy increased the risk of behavioral problems 1.5-fold in MLPs, similar to what has been reported for FTs [35,36], EPs [15,20,37], and in the only further available study that investigated this factor specifically for MLPs, though focusing on school problems [7]. A possible explanation is that maternal smoking during pregnancy leads to lower dopamine signaling, reducing response inhibition in the child [38]. Another explanation is that maternal smoking is associated with deterioration of placental function [39], increasing the risk of chronic fetal hypoxia [40], in turn causing structural brain changes associated with both internalizing [41] and externalizing [42] problems. However, our findings contrast with some findings on FTs, for whom this association has not been found [14,16]. This may be because in FT children of smoking mothers the placenta still functioned adequately, unlike in preterm born children.

We further found male gender and multiple birth to be associated with emotional and behavioral problems, both of these factors are non-modifiable, in contrast to the two factors discussed previously. For male gender we found a nearly twofold increased risk of behavioral problems, which is in line with other studies in both EPs and FTs [14,16,18], whereas for MLPs previous findings were not consistent [7,12]. This gender-specific behavior may be caused by concentrations of pre- and postnatal androgen, which are higher in males than females [43]. In our study, multiple birth, in contrast to having older siblings, decreased the risk of internalizing problems. This is a new finding. Most other studies that include having siblings at birth in their analyses do not specifically focus on being part of a twin or triplet. Huddy et al (2001) [7] have found multiparity, in contrast to multiple birth, to be associated with an increased risk of poor school performance. One explanation could be that multiples are more likely to interact intensively with each other than other children of different ages in the family, as illustrated by studies reporting more positive behaviors during play in preterm multiples compared with preterm singletons [44]. A second explanation could be that parenting for multiples is more challenging, as illustrated by studies reporting higher levels of parenting stress [44,45] and decreased parent-infant interactions [46]. It should also be kept in mind that child behavior might indirectly influence parenting behavior in accordance.

Remarkably, in our study we found only four of the 23 risk factors studied to have a significant association with emotional and behavioral problems. Regarding behavioral problems, other expected factors based on previous literature include SGA [16] and being the first-born child [17]. Regarding the full range of internalizing problems for MLPs, none of the previous studies focused on the relationship with perinatal factors. Among FTs, however, several factors have been shown to augment the risk of internalizing problems: maternal and paternal emotional problems, caesarian section, being male [14], maternal smoking, and non-Caucasian background of the mother [17]. Our community-based study in the Dutch population included a substantial number of MLPs, allowing us to conclude that for MLPs only few factors are associated with emotional and behavioral problems. Given the low number of factors associated with these problems for EPs, MLPs may have a substantial capacity for improvement with respect to the development of emotional and behavioral problems.

Our study has several strengths. Most importantly, that it is a large, community-based cohort of MLPs with a high inclusion rate, and therefore representative of the average population of MLPs in the Netherlands, underlines its clinical relevance. Additionally, we were able to analyze the effects of a large number of perinatal and social factors and assess the full range of emotional and behavioral problems.

Our study also had some limitations. First, as the CBCL is a parent-report questionnaire we relied on one of both parents’ opinions about their child’s behavior. We did not find an association between the parent completing the questionnaire and the occurrence of emotional and behavioral problems (data not shown). Although a psychiatric interview might have been more accurate, the CBCL has been shown to be highly valid in various countries, including the Netherlands [26–28]. Another limitation was that families of low SES were underrepresented in the analyses. As low SES has previously been associated with greater emotional and behavioral problems [11] this may have led to some underestimation of the actual associations. Furthermore, we included several maternal factors, however paternal factors included in our study are scarce. Since we have already evaluated a broad variety of factors in our study, and more factors are not available within our cohort study, we are unable to present more information on parental factors. Moreover, children were only included if data on all risk factors were available. This might have led to some selection bias because parents of children that are less ill will participate more often. If occurring, this may have weakened the associations as found.

Our findings contribute to the growing evidence on risk factors for emotional and behavioral problems in MLPs. This new knowledge enables PCHCs to better identify MLPs at increased risk of emotional and behavioral problems, and indicate potential targets for preventive intervention in this largest group of preterm born children. More attention is warranted for the prevention and/or treatment of two important modifiable risk factors, namely maternal smoking during pregnancy and perinatal infections. Finally, our findings warrant future studies aimed at unravelling the causal (biological) pathways between these specific perinatal factors and emotional and behavioral problems stratified for GA.

## Conclusion

Perinatal infection increased the risk of emotional and behavioral problems in MLPs at school entry. Concerning behavioral problems, perinatal infection, maternal smoking during pregnancy, as well as male gender increased the risk. Multiple birth, in contrast, decreased the risk of emotional problems in MLPs. Prevention of maternal smoking during pregnancy is thus of utmost importance. We conclude that MLPs born with a clinical perinatal infection, born from a smoking mothers, or born male should have closer monitoring during follow-up than MLPs in general.
